# Supplementary figures and images for: EBV miRNAs are potent effectors of tumor cell transcriptome remodeling in promoting immune escape
Source: PLoS Pathog. 2021 May 6;17(5):e1009217. doi: 10.1371/journal.ppat.1009217 (PMC8130916; doi:10.1371/journal.ppat.1009217)

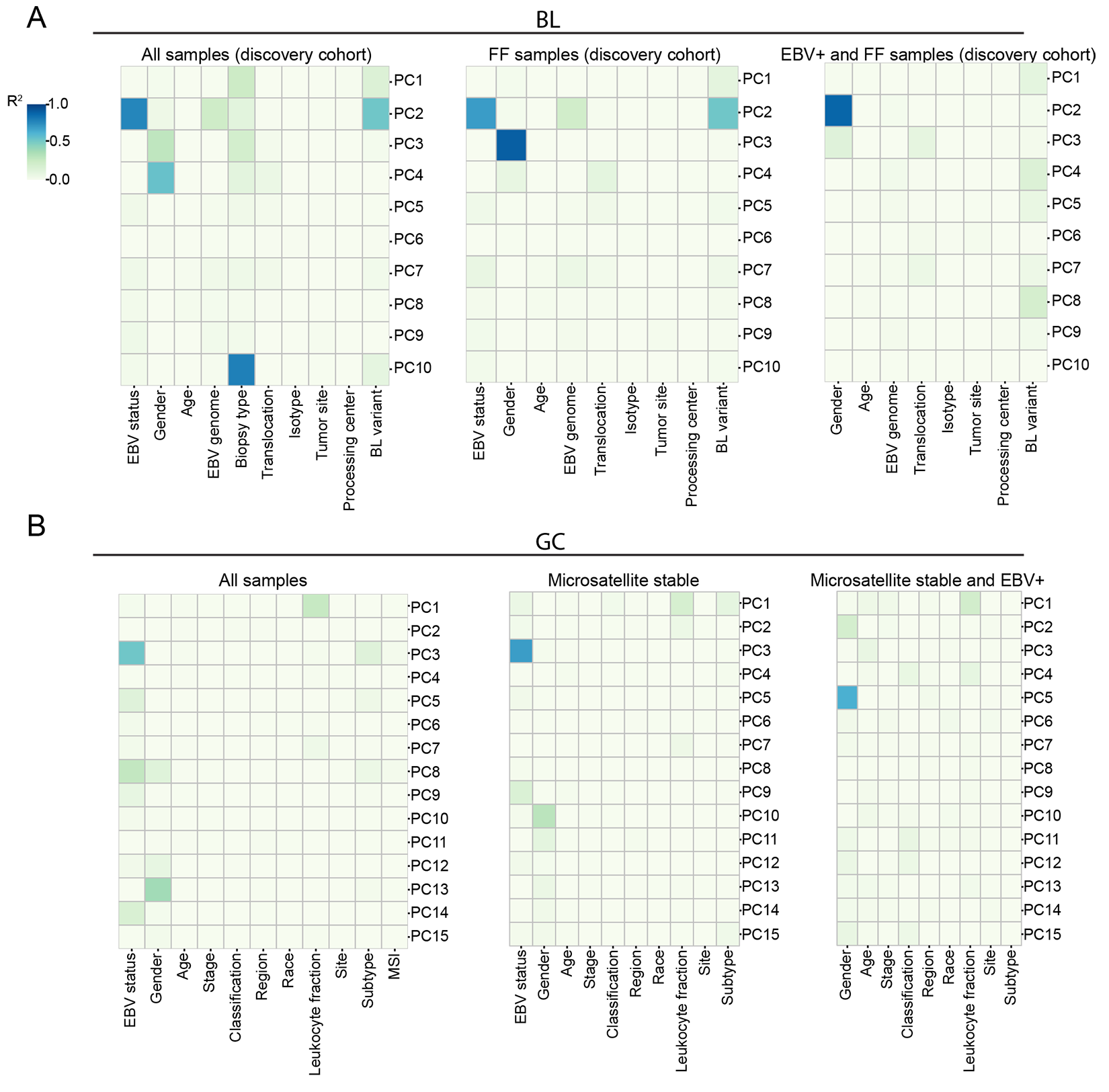

Supplement: S1 Fig — Variance stabilized BL and GC RNA expression values were subjected to principal component analysis (PCA). Logistic regression was performed comparing each of the first ten (BL) or fifteen (GC) principal components with the indicated clinical covariate[47,48,57]. The resulting R2 values from were plotted as a heatmap. (A) The BL analysis was restricted to tumors passing QC (the “Discovery” cohort)[47] (left), EBV-positive tumors of the “Discovery” cohort (center), and the EBV-positive, fresh, frozen tumors from the “Discovery” cohort (right). (B) The GC analysis included all tumors (left), exclusively microsatellite stable (MSS) tumors (center), and only MSS and EBV-positive GCs (right). (TIF) [file ppat.1009217.s001.tif]

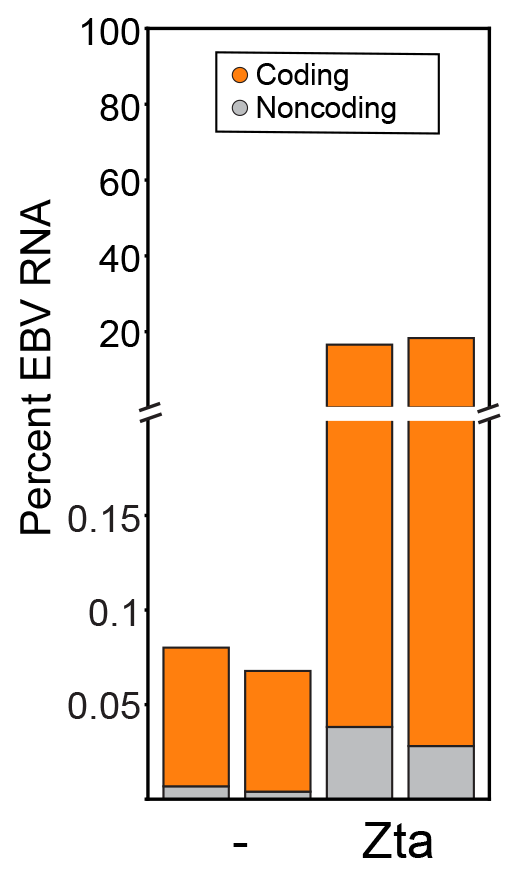

Supplement: S2 Fig — Raw sequencing reads of latent and Zta-induced Akata cells (SRA accession: SRP042043)[109] were aligned to the combined hg38 + EBV reference transcriptome using kallisto[46]. The viral percentage of expressed mRNAs was calculated using the following equation, ∑viraltranscripts(t.p.m.)106. Viral non-coding transcripts are in gray; viral coding transcripts are in orange. (TIF) [file ppat.1009217.s002.tif]

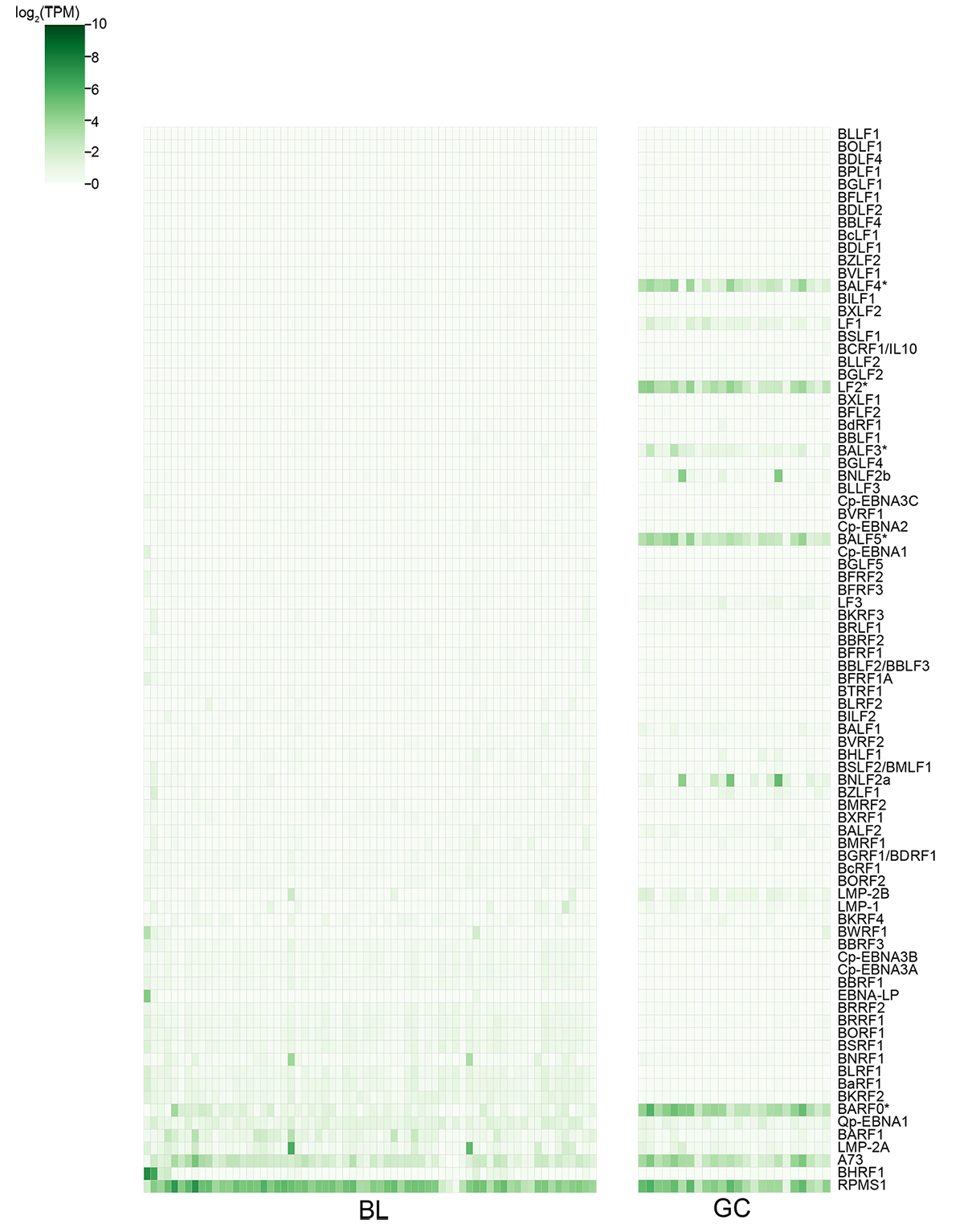

Supplement: S3 Fig — A heatmap of viral gene expression (t.p.m.) in BL and GC tumors. The heatmap does not include EBER1 or EBER2 (their sizes, 167 and 172 nucleotides, are slightly below the purified fragment size used to generate the RNA-seq libraries). RNA-sequencing of GC tumors was not strand specific and lytic genes that overlap RPMS1 and/or A73 are marked with “*” since these values likely represent misattributed RPMS1 or A73 expression. (TIF) [file ppat.1009217.s003.tif]

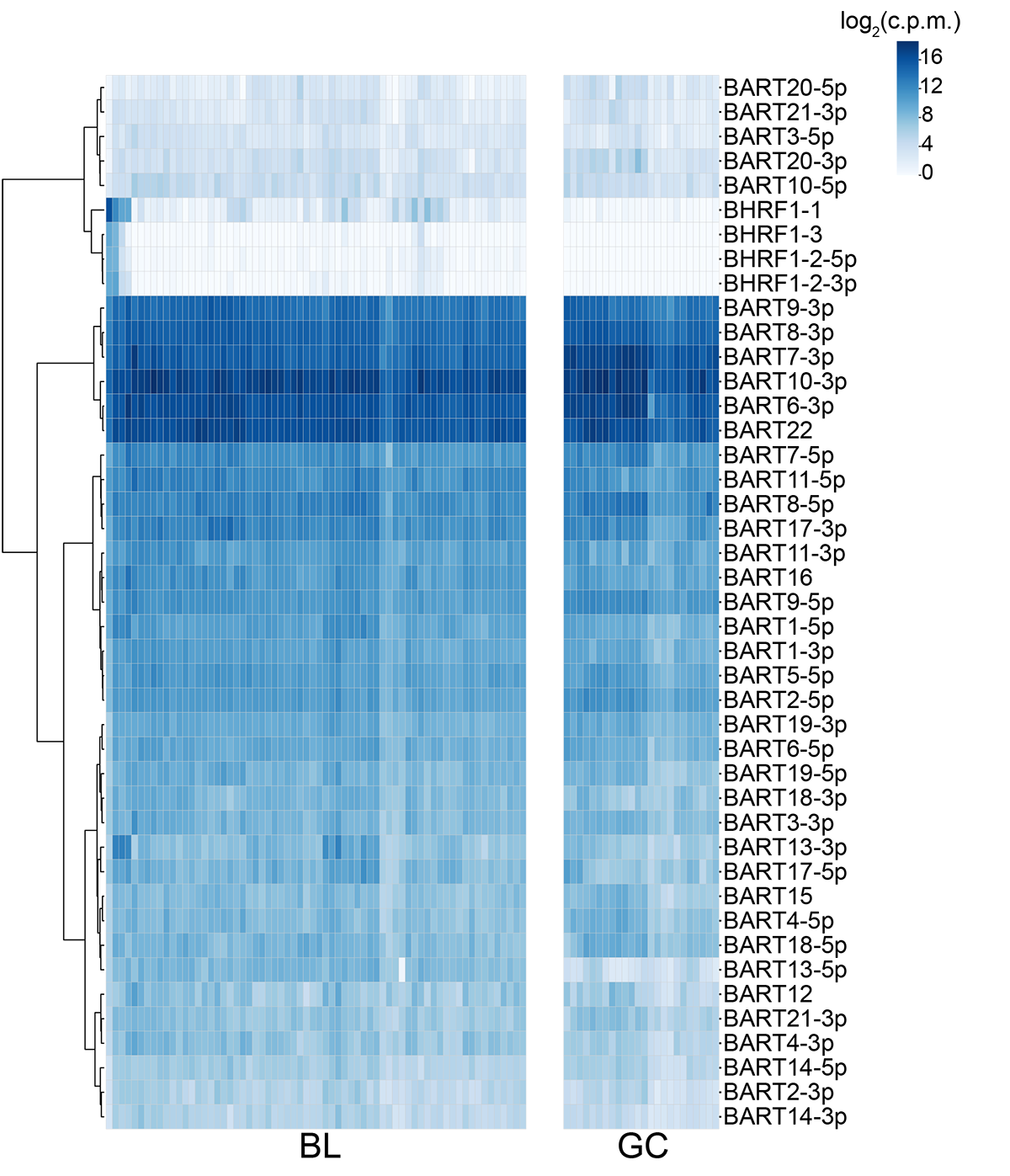

Supplement: S4 Fig — A heatmap of viral microRNA expression (c.p.m.) across all BL and GC tumors. Reads were aligned to the combined EBV and human mature microRNA references (miRBase v22) using bowtie2[110]. (TIF) [file ppat.1009217.s004.tif]

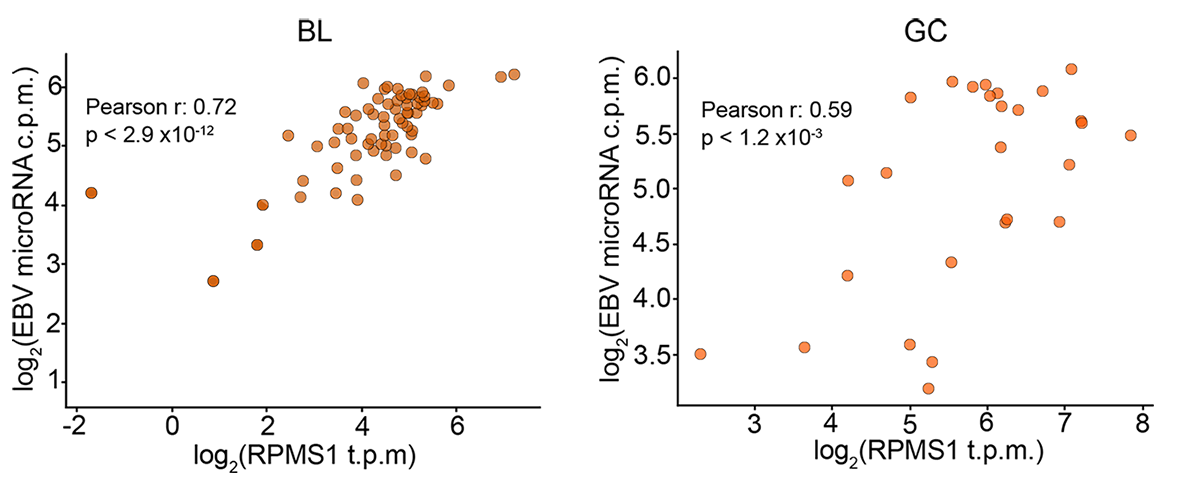

Supplement: S5 Fig — The sum of all viral microRNAs (c.p.m.) was correlated with RPMS1 expression (t.p.m.) in EBV-positive BL and GC tumors. (TIF) [file ppat.1009217.s005.tif]

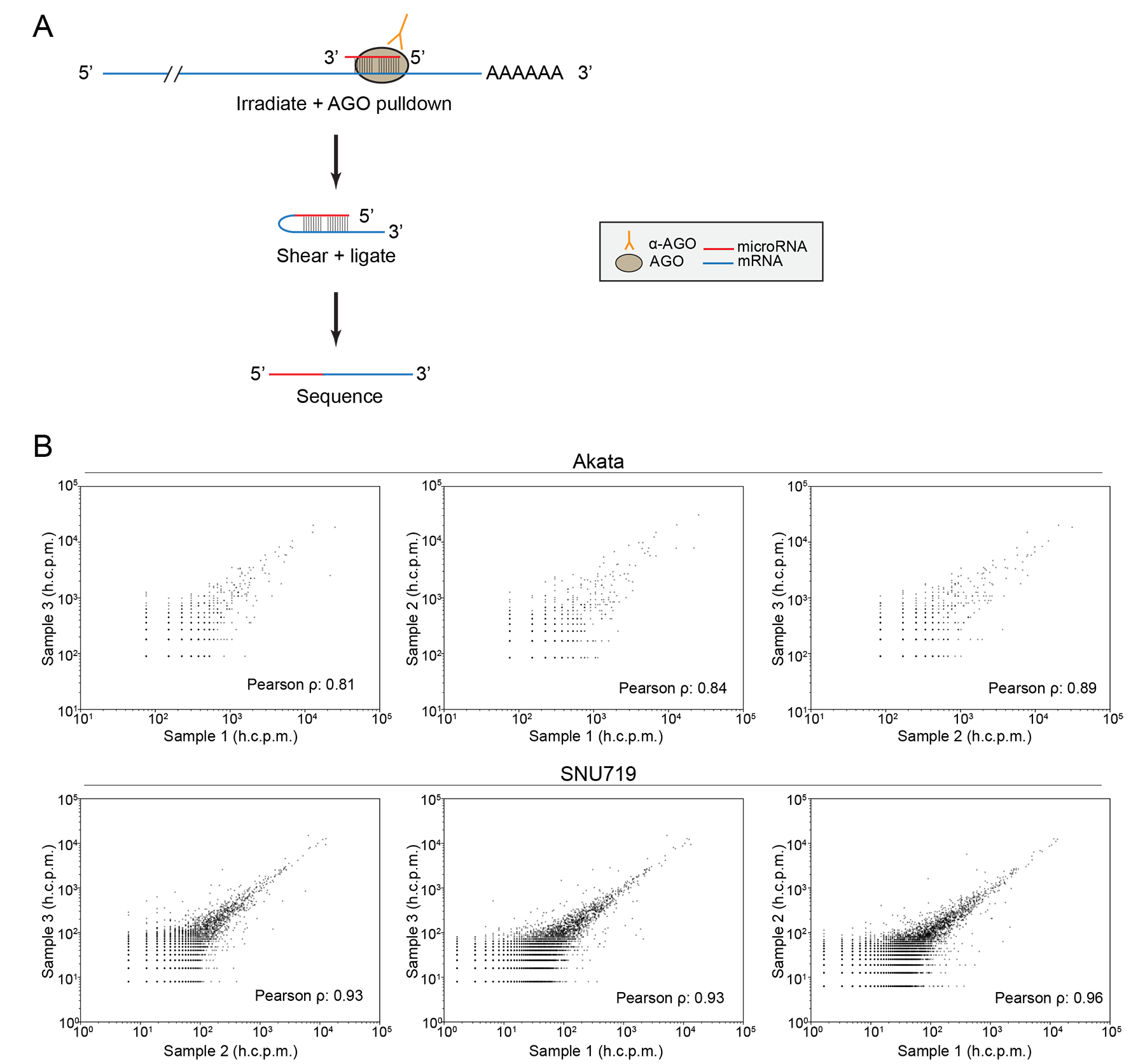

Supplement: S6 Fig — (A) Schematic of the CLASH protocol[41,42]. CLASH sequencing reads were processed using the hyb bioinformatic pipeline[111] and in house scripts (https://github.com/flemingtonlab/ebv_clash). (B) Normalized counts (hybrid counts per million, h.c.p.m.) of each microRNA-mRNA pair were correlated pairwise between replicate samples; pearson correlation coefficients are indicated. (TIF) [file ppat.1009217.s006.tif]

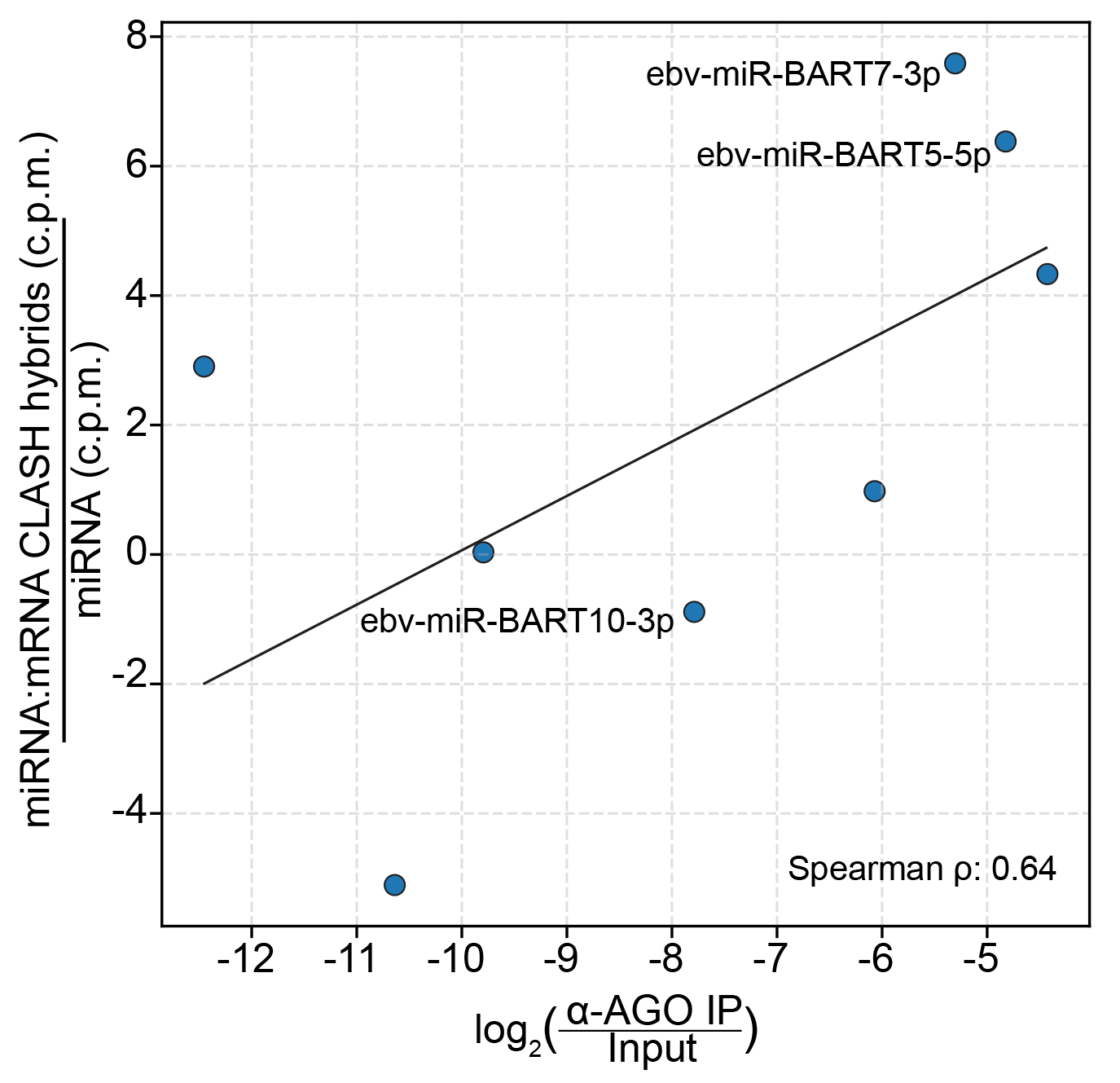

Supplement: S7 Fig — To validate the loading efficiency values obtained via CLASH and small fraction RNA-sequencing (see Fig 2), we performed an α-AGO immunoprecipitation without crosslinking or ligation, followed by qRT-PCR for several selected microRNAs. The loading efficiency for each microRNA was calculated by taking the ∂Ct between AGO-IP and input sample (2−(αAgoCt−InputCt); x-axis). These values were compared to those obtained using CLASH and small fraction RNA-seq (microRNAx:mRNA(h.c.p.m.)microRNAx(c.p.m.); y-axis), resulting in a spearman correlation coefficient of ρ = 0.64. (TIF) [file ppat.1009217.s007.tif]

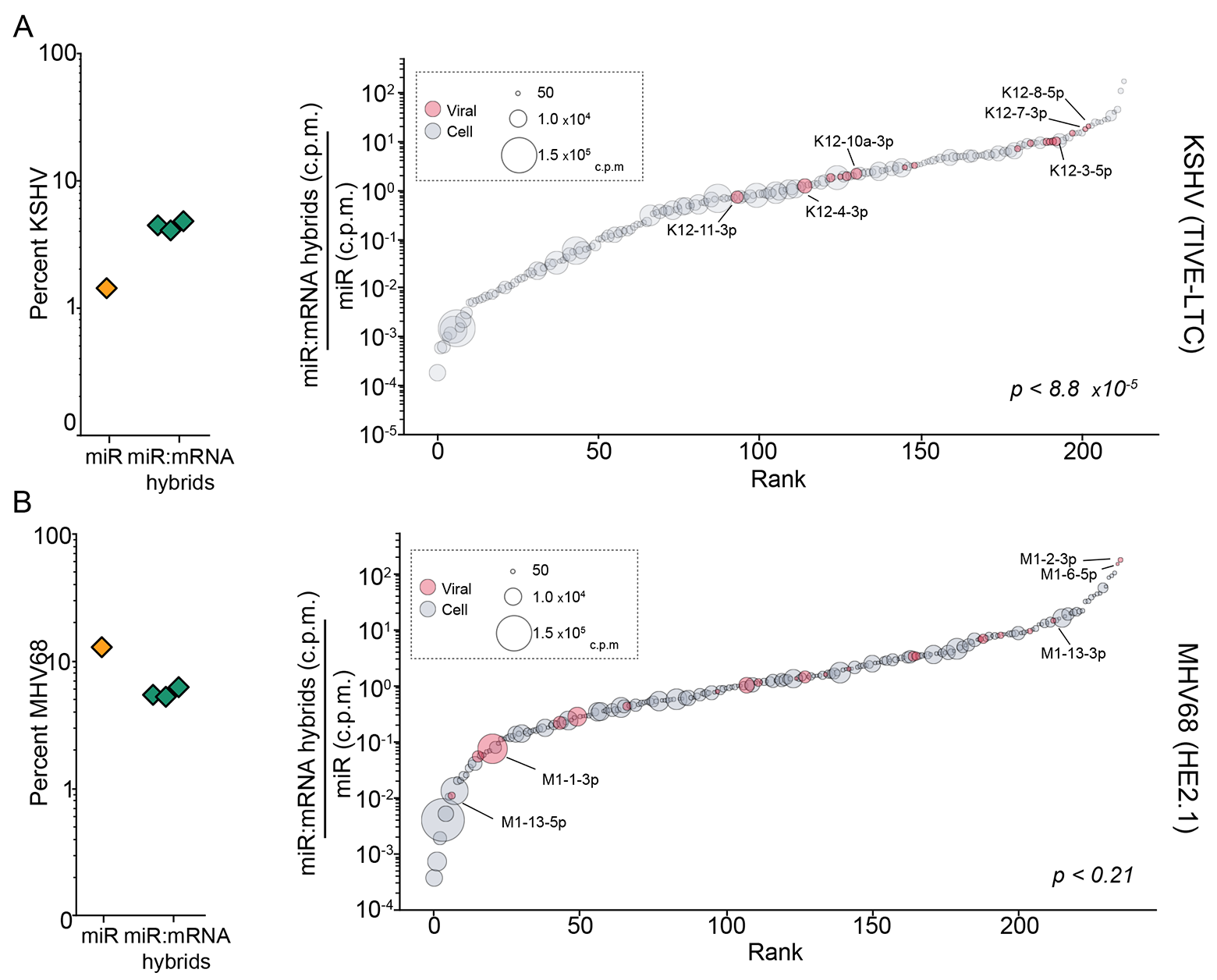

Supplement: S8 Fig — Raw CLASH sequencing reads were obtained for KSHV infected TIVE-EX-LTC[42] (SRA accessions: SRR5876950-SRR5876952) and MHV68 infected HE2.1[66] (SRA accessions: SRR8395245-SRR8395247) cell lines, with small fraction sequencing performed on the same cell lines. (A-B; left) Yellow triangles display total viral microRNA expression, ∑viralmicroRNA(c.p.m.)106; green triangles represent the percent of all microRNA-mRNA hybrids containing a viral microRNA, ∑viralmicroRNA:mRNAhybrids(h.c.p.m.)106. P-values were calculated using unpaired Student’s t-tests. (A-B; right) MicroRNA-mRNA hybrid abundance was normalized to baseline expression for each individual microRNA, microRNAx:mRNA(h.c.p.m.)microRNAx(c.p.m.) (see Fig 2). Viral and host microRNA ranks were compared; P-values were calculated using the Kolmogorov–Smirnov test (KS). (TIF) [file ppat.1009217.s008.tif]

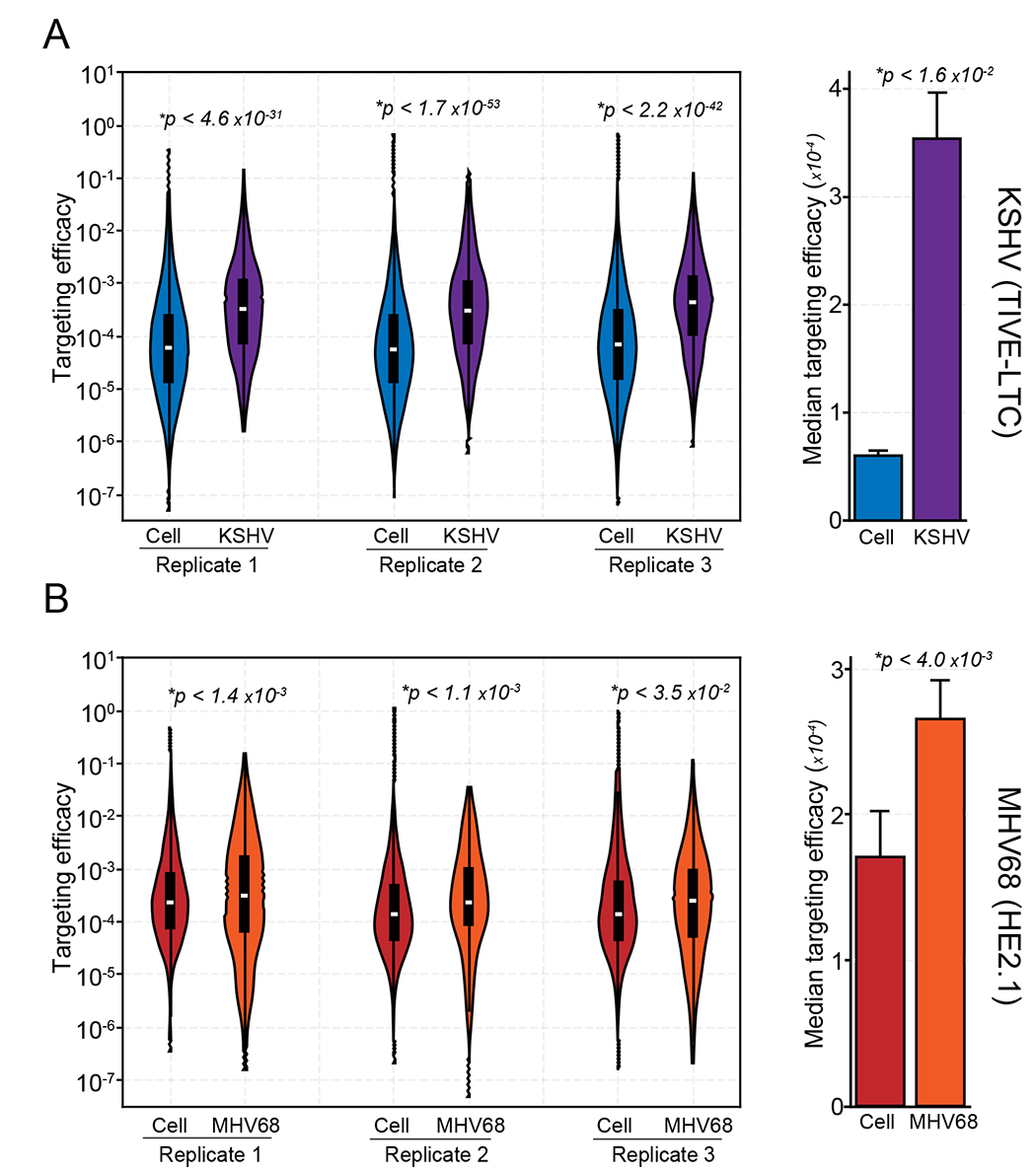

Supplement: S9 Fig — (A-B; left panels) Hybridization efficacy values were tabulated (see Fig 3B) for each CLASH replicate, comparing viral and host interactions. P-values were calculated using the Kolmogorov–Smirnov test. (A-B; right panels) The mean hybridization efficacy of viral and host hybrids from all three replicates; P-values were calculated using paired Student’s t-tests. (TIF) [file ppat.1009217.s009.tif]

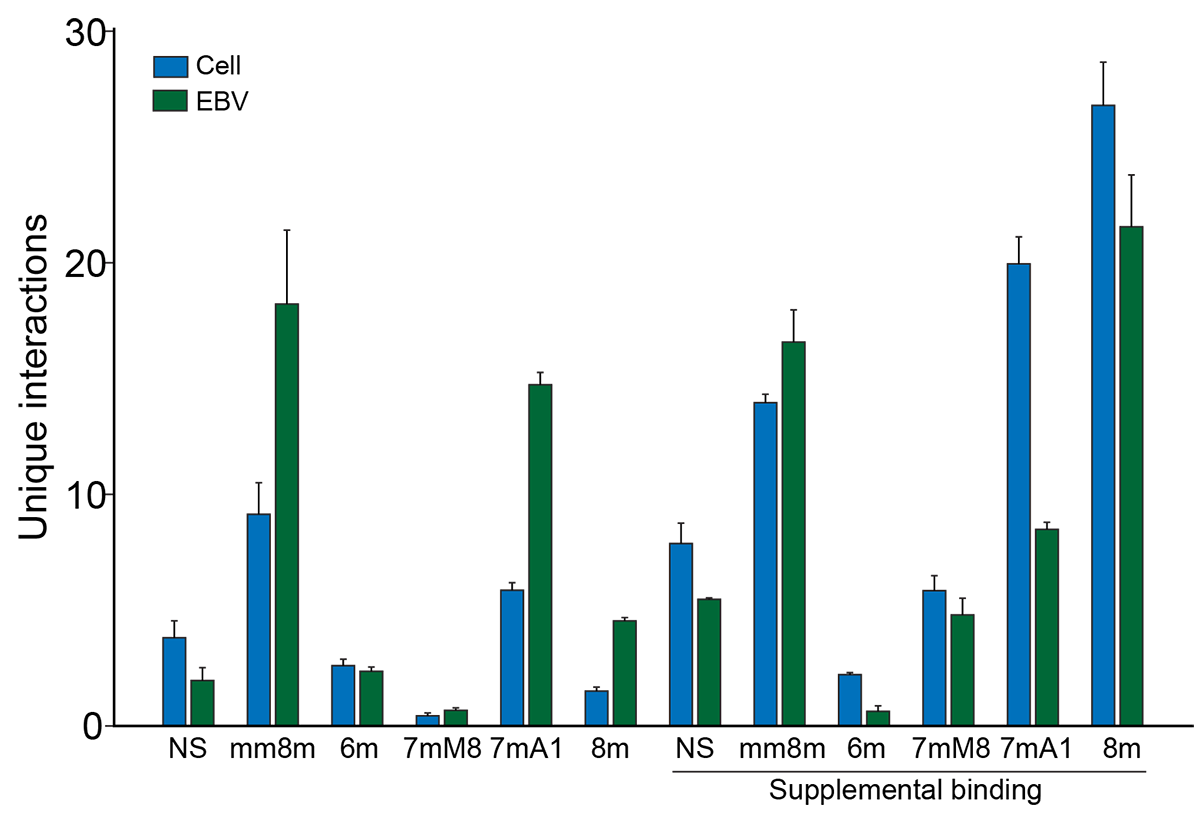

Supplement: S10 Fig — The number of unique interactions of each type of seed match were compared between viral and cellular microRNAs. (TIF) [file ppat.1009217.s010.tif]

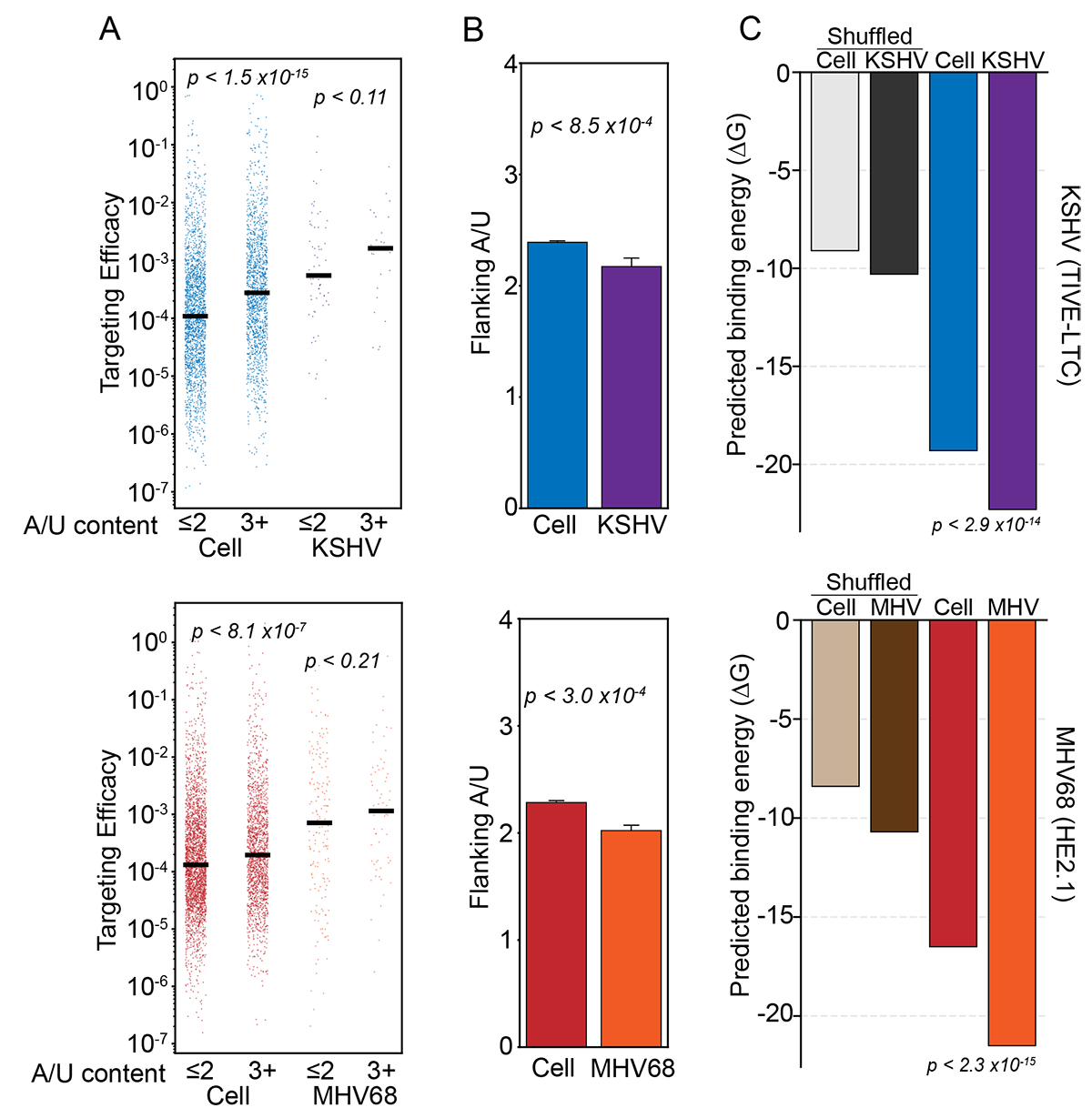

Supplement: S11 Fig — (See Fig 4). (A) The targeting efficacy of each interaction binned by flanking A/U content of each microRNA target site. P-values were calculated using the KS test. (B) The mean number of flanking A/Us (max = 4) for cellular and KSHV (top) or MHV68 (bottom) microRNA target sites. P value was calculated via KS test. (C) Predicted minimum free binding energies (ΔG) were calculated for each hybrid using the RNAcofold function of the Vienna RNA Suite[107]. As a control, ΔG calculations were performed on shuffled sequences, with 100 permutations performed for each hybrid pair. The ΔG values of cellular and viral hybrids were compared by KS test. (TIF) [file ppat.1009217.s011.tif]

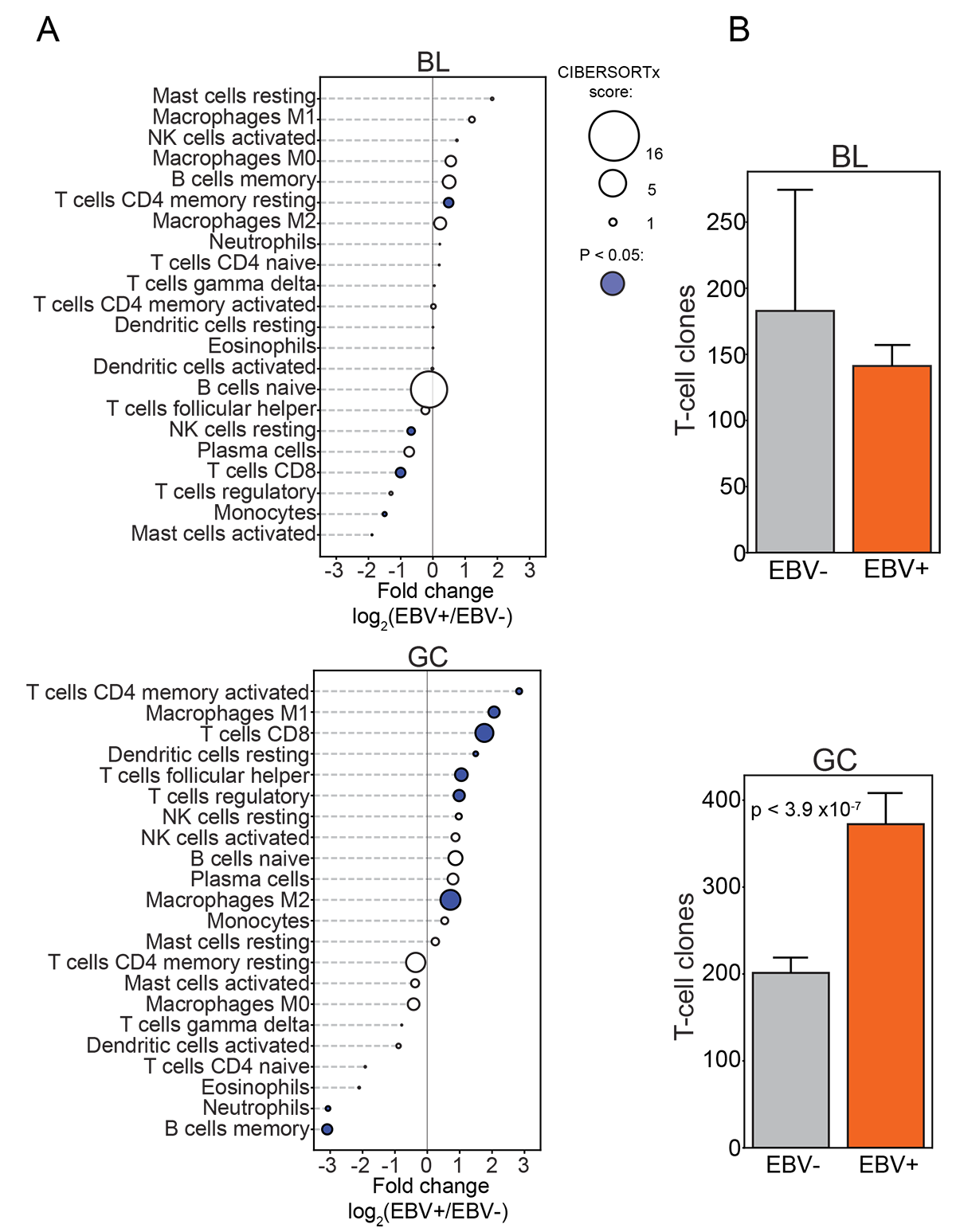

Supplement: S12 Fig — (A) Immune cell abundances were inferred using CIBERSORTx. Tumor immune cell compositions were compared between EBV-positive and EBV-negative BL and GC tumors. Circle size represents the average CIBERSORTx absolute score across all tumors, filled circles represent statistically significant (P < 0.05) fold changes; P-values were calculated using Mann Whitney U tests. (B) The number of unique T-cell clonotypes was compared between EBV-positive and EBV-negative BL and GC tumors. P-values were calculated using unpaired Student’s t-tests. (TIF) [file ppat.1009217.s012.tif]

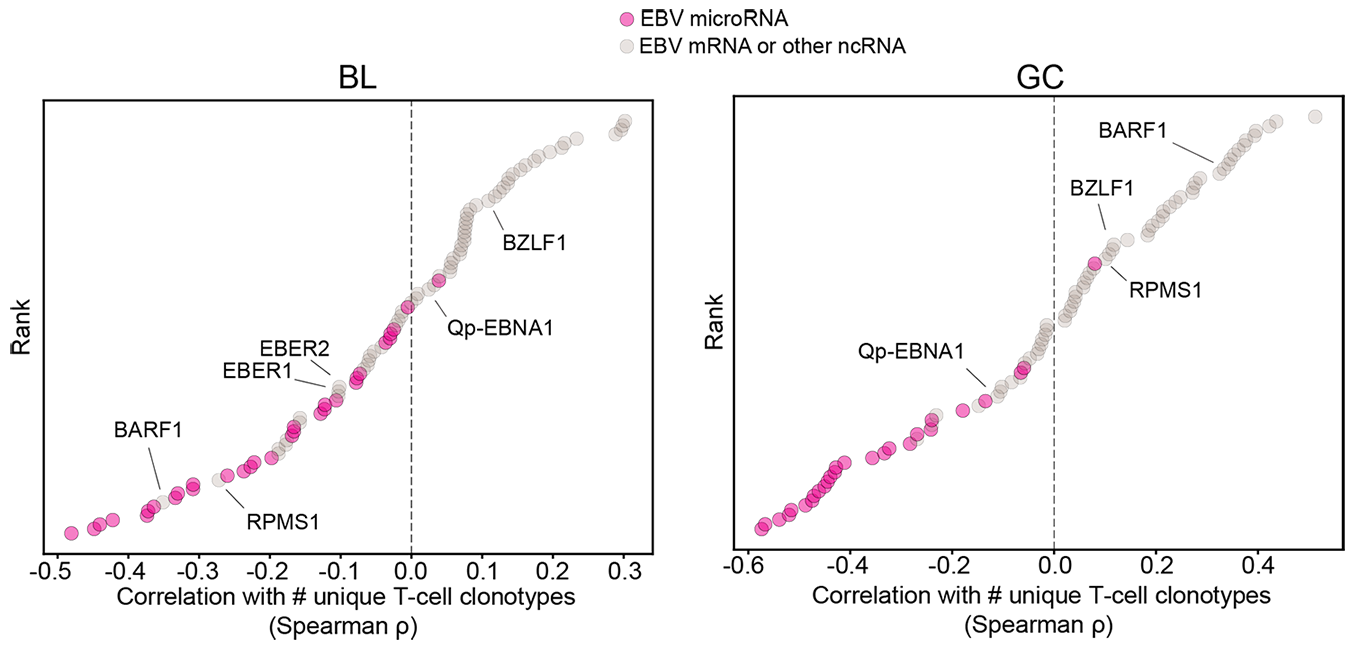

Supplement: S13 Fig — Expression values of each EBV lncRNA, mRNA, and microRNA was correlated with counts of unique T-cell clonotypes in EBV-positive BL and GC tumors. Spearman correlation coefficients were plotted in ranked order. (TIF) [file ppat.1009217.s013.tif]
